# The effect of decreasing permafrost stability on ecosystem carbon in the northeastern margin of the Qinghai–Tibet Plateau

**DOI:** 10.1038/s41598-018-22468-6

**Published:** 2018-03-08

**Authors:** Wenjie Liu, Shengyun Chen, Junyi Liang, Xiang Qin, Shichang Kang, Jiawen Ren, Dahe Qin

**Affiliations:** 1 Institute of Tropical Agriculture and Forestry, Hainan University, Haikou, 570228 China; 2 Qilian Shan Station of Glaciology and Ecologic Environment, State Key Laboratory of Cryospheric Science, Northwest Institute of Eco-Environment and Resources, Chinese Academy of Sciences, Lanzhou, 730000 China; 3 Environmental Science Division and Climate Change Science Institute, Oak Ridge National Laboratory, Oak Ridge, Tennessee 37831 USA

## Abstract

The objective of this study is to investigate the effect of decreased permafrost stability on carbon storage of the alpine ecosystems in the northeastern margin of the Qinghai–Tibet Plateau. During July and August 2013, we selected 18 sites in five types of permafrost (stable, substable, transitional, unstable, and extremely unstable) regions. We measured aboveground phytomass carbon (APC) and soil respiration (SR), soil inorganic carbon (SIC), soil organic carbon (SOC), belowground phytomass carbon, and soil properties down to 50 cm at same types of soils and grasslands. The results indicated that ecosystem carbon in cold calcic soils first decreased and then increased as the permafrost stability declined. Overall, decreasing permafrost stability was expected to reduce ecosystem carbon in meadows, but it was not obvious in swamp meadows and steppes. APC decreased significantly, but SIC and SOC in steppes first decreased and then increased with declining permafrost stability. Soil clay fraction and soil moisture were the controls for site variations of ecosystem carbon. The spatial variations in SR were possibly controlled by soil moisture and precipitation. This meant that alpine ecosystems carbon reduction was strongly affected by permafrost degradation in meadows, but the effects were complex in swamp meadows and steppes.

## Introduction

Permafrost plays a fundamental role in alpine ecosystems, and permafrost degradation has been shown to have significant consequences for these ecosystems^1–3^. In recent years, arctic and subarctic climate warming has significantly enhanced permafrost degradation^4–9^, expansion of shrub vegetation communities, hydrological cycles, and CO_2_ and CH_4_ emissions. These are associated with the loss of large amounts of soil carbon stored in permafrost soils at high-latitudes in the Northern Hemisphere^4,10–13^. Similarly, the effects of warming on permafrost, vegetation, and soil nutrients in association with soil carbon reduction also occurred in high-altitude regions^2,5,14–16^. Quantifying these changes is important for evaluating the global terrestrial carbon cycle and atmospheric carbon budget, and related climate system feedback processes.

The Qinghai–Tibet Plateau (QTP) is the largest high-altitude permafrost region on Earth. The QTP’s permafrost region contains ~67.2 Pg of soil organic carbon (SOC), and SOC storage in the 0–1 m depth range was ~15.29 Pg^17^. Recent studies have determined that the active layer thickness of permafrost in the QTP has increased over the past several decades because of global warming^5,6,8,18,19^. In addition, a significant reduction in permafrost area has also occurred, and only ~1.1 × 10^6^ km^2^ of the permafrost area remains according to the most recent report in 2016^20^. Results from a recent simulation suggest that areas of permafrost on the QTP will further decrease^14^. Permafrost degradation profoundly affects alpine ecosystems, thus leading to decreased grassland coverage and biological production^15^, a low water table, modifications to surface and subsurface hydrology^21^, altered vegetation growth and soil nutrient cycling^5,22^, and changes in energy and carbon exchange between the soil and atmosphere^5^. The dominant ecosystems on the QTP are alpine grasslands (alpine steppes and alpine meadows), which occupy more than 60% of the total area^23,24^. In addition to overgrazing, this reduction in permafrost has led to considerable losses of carbon from heavily degraded alpine grasslands, thus resulting in significant modifications to the regional carbon cycle^25,26^. Examining the patterns and factors controlling permafrost degradation is essential to gain an appropriate understanding of the characteristics associated with different forms of carbon and the manner in which they are regulated in permafrost regions.

Soil respiration (SR) is the primary pathway by which CO_2_ fixed by land plants returns to the atmosphere^27^, and it plays a central role in belowground carbon cycling. Permafrost regions have a large pool of soil carbon; thus, small changes in SR may have large effects on atmospheric CO_2_ concentrations, thus creating a positive feedback in global warming processes^28^. SR is primarily influenced by microbial activity, the amount and composition of soil carbon, and regulating factors, such as climate (air temperature and precipitation) and soil variables (soil temperature, moisture, and texture). Because the dominant factors differ among different ecosystems, SR may vary significantly as a function of time and location^29^. Temporal variations in SR have been observed at a single site using advanced equipment for high-frequency measurements^30^. Spatial variations in SR have also been the focus of many studies^14,30,31^. A comprehensive understanding of spatial variations in SR in permafrost regions would provide a valuable foundation for studying regional carbon cycles and their responses to permafrost degradation and climate change.

The upper reaches of the Shule River Basin are in the northeastern margin of the QTP, where large quantities of high-altitude permafrost occur^32^. Over the past 20 years, large areas of permafrost in this region have experienced serious degradation due to overgrazing and global warming, which has had a significant impact on hydrological processes^33^, soil environment and vegetation composition^13,34^. Previous studies have indicated that alpine grassland would respond differently to permafrost degradation in different types of permafrost zones^35^. Changes in aboveground and belowground components caused by vegetation succession from alpine swamp meadows to alpine meadows and steppes have indirectly or directly resulted in a loss of ecosystem carbon stock^36^. In addition, more than 90% of belowground biomass in alpine grasslands has been shown to be concentrated in the top 30 cm of soil^37^, and SOC is concentrated in topsoil and correlates well with BPC in this area^38^. For those reasons, ecosystem carbon above ground and below ground at a 0–50 cm depth range, like soil carbon in permafrost, were easily influenced by permafrost degradation. In this study, we selected several sites in different permafrost zones with the same soil or grassland vegetation types to conduct a systematic investigation of SR, vegetation, and soil properties at a depth of 0–50 cm.

The objectives of this article are to (1) assess the effects of permafrost degradation on ecosystem carbon (EC), SOC, soil inorganic carbon (SIC), belowground phytomass carbon (BPC), aboveground phytomass carbon (APC), and SR; and (2) examine the effects of environmental factors on spatial patterns of EC, APC, BPC, SOC, and SIC, and SR. Unlike previous assessments of the effect of permafrost degradation on SOC in the QTP, our new estimation focuses on the same soil or grassland types and includes different forms of carbon. The results of this study can provide critical data needed to model the effects of permafrost degradation on carbon cycling in the QTP.

## Results

### Different forms of carbon density distributions as a function of soil or grassland type

**Figure 1:**
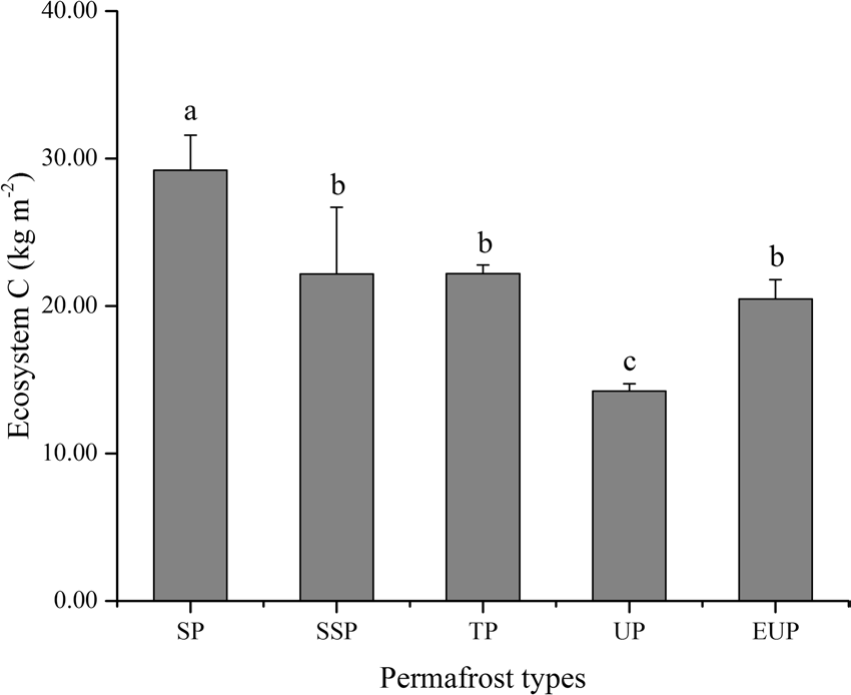
Variations in ecosystem carbon (EC) among different permafrost zones in cold calcic soils (SP: stable permafrost; SSP: substable permafrost; TP: transitional permafrost; UP: unstable permafrost; EUP: extremely unstable permafrost). Error bars express standard deviation from the mean. Different permafrost zones show significant differences when columns are marked with different letters and non-significant differences when columns are marked with same letters. The figure was generated using OriginPro 2016 (64-bit) b9.3.226 (http://www.originlab.com/).

Cold calcic soil was distributed in five different permafrost stability zones (Table S1), which were selected to evaluate the effects of permafrost degradation on different forms of carbon. The EC in cold calcic soils in different permafrost zones are shown in Fig. 1, and the highest and lowest amounts of EC were found instable and unstable permafrost zones, respectively. The SIC, SOC, the sum of SIC and SOC (SC), and BPC at depths of 0–50 cm, APC, and total phytomass carbon (PC) are shown in Fig. S1. No obvious patterns in SIC, SOC, or SC variation were observed with decreasing permafrost stability.

**Figure 2:**
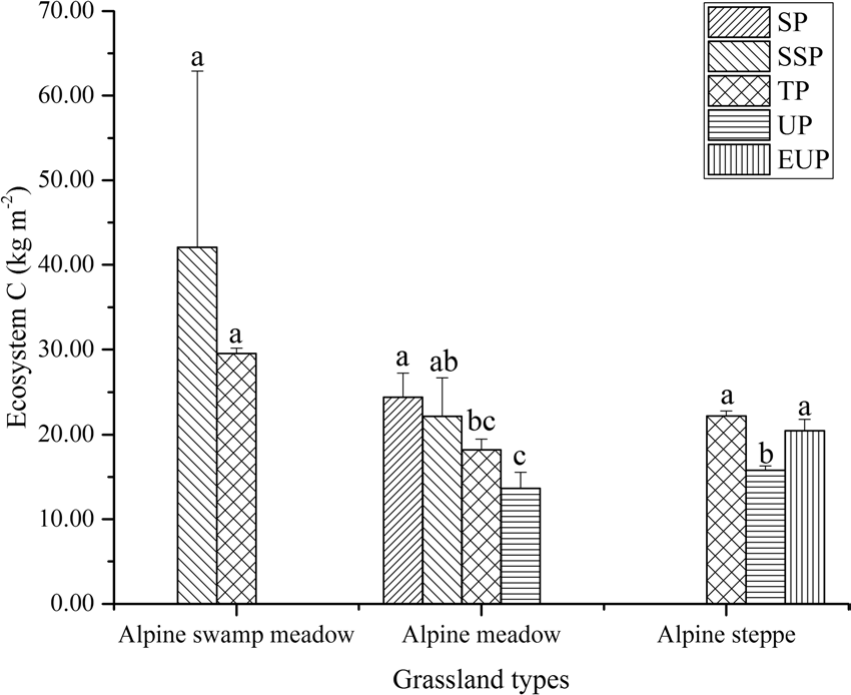
Variations in ecosystem carbon among different permafrost zones in alpine swamp meadows, alpine meadows, and alpine steppes (SP: stable permafrost; SSP: substable permafrost; TP: transitional permafrost; UP: unstable permafrost; EUP: extremely unstable permafrost). Error bars express standard deviation from the mean. Within one grassland type, different permafrost zones show significant differences of one form carbon when columns are marked with different letters and non-significant differences of one form carbon when columns are marked with same letters. The figure was generated using OriginPro 2016 (64-bit) b9.3.226 (http://www.originlab.com/).

**Figure 3:**
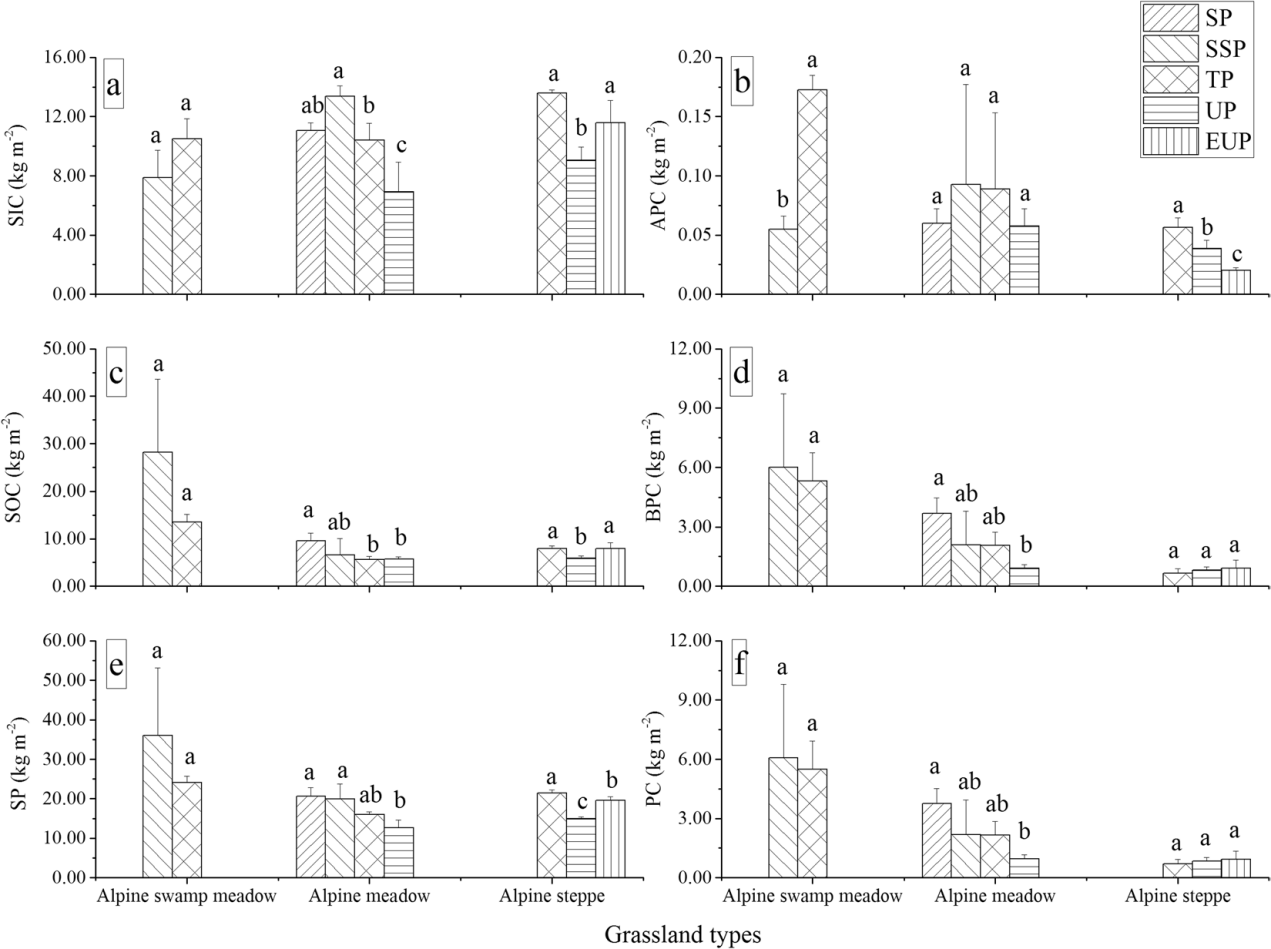
Variations in soil inorganic carbon (SIC), soil organic carbon (SOC), soil carbon (SC, the sum of SIC and SOC), and belowground phytomass carbon (BPC) at depths of 0–50 cm, along with aboveground phytomass carbon (APC) and the sum of APC and BPC (PC) among different permafrost zones in alpine swamp meadows, alpine meadows, and alpine steppes. Legend key is similar to that in Fig. 3. Error bars express standard deviation from the mean. Within one grassland type, different permafrost zones show significant differences of one form carbon when columns are marked with different letters and non-significant differences of one form carbon when columns are marked with the same letters. The figures were generated using OriginPro 2016 (64-bit) b9.3.226 (http://www.originlab.com/).

The variations in the EC among different permafrost zones in alpine swamp meadows, alpine meadows, and alpine steppes are shown in Fig. 2. For alpine swamp meadows, there was no significant difference in EC between the substable and transitional permafrost zones. The EC gradually decreased in alpine meadows from the stable permafrost zone to the unstable permafrost zone. The EC first decreased and then increased in alpine steppes from transitional permafrost to extremely unstable permafrost zones. Similarly, Fig. 3 shows the SIC, SOC, and SC at depths of 0–50 cm among different permafrost zones in alpine swamp meadows, alpine meadows, and alpine steppes. Alpine swamp meadows exhibited no significant differences in SIC, SOC, or SC between the substable and transitional permafrost zones. Although the highest SIC values in alpine meadows occurred in the substable permafrost zone, SOC and SC decreased slightly as the permafrost stability declined from stable to unstable. In alpine steppes, SIC, SOC, and SC first decreased and then increased from the transitional to the extremely unstable permafrost zones.

Figure 3 also shows variations in APC, BPC, and total phytomass carbon (PC, the sum of APC and BPC) in alpine swamp meadows, alpine meadows, and alpine steppes among different permafrost zones. In alpine swamp meadows, APC in the substable permafrost zone were significantly lower than those in the transitional permafrost zone; however, no significant differences BPC occurred at depths of 0–50 cm. There were no obvious changes in APC in alpine meadows, but BPC decreased gradually with decreasing permafrost stability from stable to unstable. The APC densities in alpine steppes decreased significantly, while there were no significant variations in the BPC from the transitional to the extremely unstable permafrost zones.

### Influence factors associated with site spatial variations in densities of different carbon forms

Among the site variables tested, the results of correlation (Pearson correlation coefficients) and linear regression analysis between different carbon densities at depths of 0–50 cm and environmental factors are displayed in Tables 1 and 2. In addition, the results of a one-sample Kolmogorov–Smirnov test for variables showed that all variables had normal distributions (Table S2). Stepwise linear regression analysis indicated a significant positive correlation between the soil clay fraction and SIC, which could explain 36.6% of the SIC variation. Significant positive correlations were found between total nitrogen (TN) and BPC with SOC; however, soil moisture (SM) values accounted for the greatest amount of variation in SOC according to the stepwise linear regression analysis. SM exhibited a significant positive correlation with APC, BPC, and EC. TN was positively correlated with BPC or EC. The stepwise linear regression analysis indicated that SM could explain the greatest amount of variation in APC and BPC, and SM along with soil clay fraction could explain the greatest amount of variation in EC (Table 2).

**Table 1:** Correlation matrix (Pearson correlation coefficients) for variables in 18 sites (soil parameter dataset from 0–50 cm) (n = 18, excluding SR).

	SIC (kg·m^−2^)	SOC (kg·m^−2^)	APC (kg·m^−2^)	BPC (kg·m^−2^)	EC (kg·m^−2^)	TN (kg·m^−2^)	SM (%)	Ecb (mS m^−1^)	ST (°C)	Clay (%)	MAAT (°C)	MAP (mm)
SIC (kg·m^−2^)	1											
SOC (kg·m^−2^)	−0.01	1										
APC (kg·m^−2^)	0.17	0.22	1									
BPC (kg·m^−2^)	−0.08	0.79**	0.57*	1								
EC (kg·m^−2^)	0.41	0.89**	0.38	0.77*	1							
TN (kg·m^−2^)	0.04	0.98**	0.20	0.74*	0.89**	1						
SM (%)	0.08	0.40	0.78*	0.73**	0.50*	0.41	1					
Ecb (mS m^−1^)	0.44	0.07	−0.23	−0.16	0.20	0.13	−0.07	1				
ST (°C)	0.04	−0.33	−0.25	−0.25	−0.27	−0.36	−0.27	0.34	1			
Clay (%)	0.61**	−0.20	0.23	0.25	0.35	0.39	-0.15	0.80**	0.06	1		
MAAT (°C)	0.46	−0.30	−0.07	−0.35	−0.10	−0.30	−0.40	0.42	0.69**	0.45	1	
MAP (mm)	−0.45	0.32	0.07	0.42	0.14	0.35	0.42	−0.37	−0.61**	−0.31	−0.90**	1
SR (n = 12) (μmol m^−2^ s^−1^)	−0.55	0.27	0.50	0.64*	−0.03	0.30	0.73**	−0.31	−0.25	0.39	−0.66*	0.79**

SIC: Soil inorganic carbon; SOC: soil organic carbon; APC: aboveground phytomass carbon; BPC: belowground phytomass carbon; EC: ecosystem carbon; TN: total nitrogen; SM: soil moisture; Ecb: bulk electrical conductivity; ST: soil temperature; Clay: soil clay fraction; MAAT: mean annual air temperature; MAP: mean annual precipitation; SR: soil respiration.

**Table 2:** Results from stepwise linear regressions of the different forms of carbon densities and soil temperature (ST), soil moisture (SM), soil clay fraction (Clay), mean annual air temperature (MAAT), and mean annual precipitation (MAP) for 18 sampling sites.

Depth (cm)	Linear Equation	*R*	*P*-value
0–50 cm	SIC = 0.350Clay + 5.529	*R* = 0.605	<0.05
	SOC = 0.221SM + 2.029	*R* = 0.740	<0.01
	BPC = 0.156SM − 1.878	*R* = 0.835	<0.01
	APC = 0.003SM + 0.005	*R* = 0.768	<0.01
	EC = 0.413SM + 0.494Clay + 2.843	*R* = 0.799	<0.01

SIC: Soil inorganic carbon; SOC: soil organic carbon; APC: aboveground phytomass carbon; BPC: belowground phytomass carbon; EC: ecosystem carbon; Values of *P* < 0.05 were considered to be significant.

### Soil respiration and contributing factors

Across 12 sites, the daily mean SR for soils with plants in a vigorous growth stage was 2.82 μmol CO_2_ m^−2^ s^−1^, with values ranging from 0.45 to 5.81 μmol CO_2_ m^−2^ s^−1^ (Fig. S2), and a coefficient of variation of 56%. Diurnal variations in CO_2_ emission rates for alpine meadow grasslands at sites SLP2 and SLP4 are shown in Fig. S3. Although measurements were conducted for three or four days, we selected the one-day variations of SLP2 and SLP4 as representative values for the entire field season (July–August 2013). We observed large diurnal variations in SR; however, the diurnal patterns generally resembled a sinusoidal curve. The highest SR values in this study occurred from 16:00 to 18:00 Beijing standard time (BST), and the lowest SR values occurred from 6:00 to 8:00 BST.

**Figure 4:**
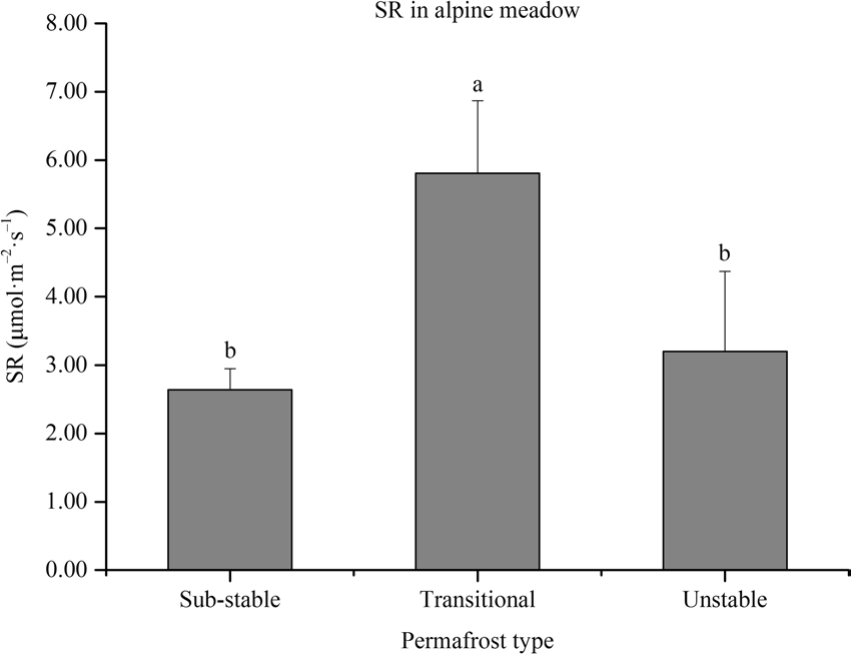
Variations in soil respiration (SR) among different permafrost zones in alpine meadows. Error bars express the standard deviation from the mean. Different permafrost zones show significant differences of SR when columns are marked with different letters and non-significant differences of SR when columns are marked with the same letters. The figure was generated using OriginPro 2016 (64-bit) b9.3.226 (http://www.originlab.com/).

Figure S4 shows the SR in cold calcic soils; the highest and lowest SR values were observed in the unstable and transitional permafrost zones, respectively. The SR in alpine meadows and alpine steppes are shown in Fig. 4 and S5, respectively. The SR in alpine meadows first increased and then decreased from the substable to unstable permafrost zone, while the SR in alpine steppes first increased and then decreased from the transitional to extremely unstable permafrost zones. Significant positive correlations were found between BPC, SM, and MAP with SR, but significant negative correlations were found between mean annual air temperature (MAAT) with SR. In addition, mean annual precipitation (MAP) accounted for most of the spatial variation in SR according to the stepwise linear regression analysis (SR = 0.010 MAP–0.445, *R* = 0.790, *P* = 0.002). If MAP is not considered, SM accounted for most of the spatial variation in SR (SR = 0.126SM–0.043, *R* = 0.726, *P* = 0.008).

## Discussion

### Response of different forms C to declines in permafrost stability

Permafrost degradation can lead to significant changes to ecosystems. For forests on ice-rich permafrost in Alaska, thawing permafrost can lead to the wholesale conversion of ecosystems from terrestrial to aquatic or wetland systems and can severely impact upland sites^39^. A previous study indicated that increased litter abundance results from summer warming in the Arctic, reflecting the increased abundance of evergreen trees and low-growing and tall shrubs^40^. However, the effects permafrost degradation on grasslands in the QTP showed other features. Permafrost degradation will be accompanied by progressive drying of the surface (surface soil materials become coarse and gravelly) and a decrease in vegetation cover and biomass in grasslands of the central QTP^5,15^. EC in the upper reaches of the Shule River Basin gradually decreased in alpine meadows from the stable permafrost zone to the unstable permafrost zone (Fig. 2). SM and soil clay fraction were the controls for EC from the stepwise linear regression result (Table 2). Permafrost degradation patterns have direct and indirect impacts on SM conditions, and soil texture is a critical factor for water retention, e.g., sandy soils have low available water capacity and silty-clay substrates have a high potential to hold water^38^. With the decrease of SM, species composition was relatively stable and weeds had difficulty invading in alpine meadows with high coverage^13^; thus, variations in SC and PC gradually decreased as permafrost stability declined (Fig. 3).

The EC in alpine steppes first decreased and then increased from the transitional permafrost zone to the extremely unstable permafrost zone (Fig. 2), which can be attributed to changes in SOC and SIC (see discussion below), because they occupied the larges proportions of EC. The APC in alpine steppes decreased significantly, while the BPC showed no obvious decreases and even increased slightly from the transitional permafrost zone to the extremely unstable permafrost zone (Fig. 3). Potential explanations for the phytomass changes include the following: (1) the coverage of alpine steppes was relatively low (30–40%) in this region with continental arid desert climate, and the high aboveground phytomass weeds (e.g., *Artemisia nanschanica Krasch*, *Leontopodium japonicum*) invading alpine steppes could not decrease (slight increase) in the total APC; (2) under the condition being limited by SM in alpine steppe, plants would concentrate on underground roots^41^.

The average active layer thickness is thick (from 1.5 to 3.9 m) in the study area (Table S1). The effects of declining permafrost stability on EC are synthetical effects of variables of permafrost, temperature, precipitation, soil properties, vegetation, and so on. Within the framework of understanding global climate warming processes and declining permafrost stability, quantifying the relationships between EC and the influencing factors investigated in this study could provide fundamental data for use in predicting changes in carbon storage on the study area and QTP. We found significant positive correlations between BPC with SOC at depths of 0–50 cm, which indicates that BPC was likely the main source and dominant influence of SOC density in the upper reaches of the Shule River Basin. This is consistent with the results of other studies that identify plant roots as a major source of SOC^38,42–44^. In addition, the stepwise linear regression analysis results show that SM accounts for the greatest amount of spatial variation in BPC (Table 2). These findings suggest that SM plays a critical role in determining the spatial distribution of BPC and SOC density in permafrost regions of the QTP, and that SM may be an effective variable for predicting BPC and SOC density across large-scale regions, such as in the central region of the QTP^23^, across China^45^, and on a global scale^44^. Permafrost partially plays a fundamental role in the current QTP ecosystems by providing moisture for plant growth^38^. The majority of net primary production (70%) by vegetation in alpine ecosystems occurs in the belowground portions of plants^46^. Our previous results also revealed that the average ratio of belowground phytomass to aboveground phytomass is 32 in the study area and that belowground components of vegetation are an important source of SOC^38^. Thus, BPC will have a strong influence on EC with declining permafrost stability.

### Changes of SIC and controls with declines in permafrost stability

Although the highest SIC values in alpine meadow grasslands were observed in the substable permafrost zone, SIC decreased as the permafrost stability declined from stable to unstable (Fig. 3). In alpine steppes, SIC first decreased and then increased from transitional to extremely unstable permafrost zones. The SIC values at depths of 0–50 cm in alpine steppe in the unstable permafrost zone were lower than those in the transitional and extremely unstable permafrost zones (Fig. 3). The soil electrical conductivity at depths of 0–50 cm in alpine steppes in the unstable permafrost zone was lower than that in the transitional and extremely unstable permafrost zones (Fig. S6), thus indicating that soil salinization was one of the potential factors that influenced SIC. Formation of secondary carbonates in soils is an effective carbon sequestration mechanism for SIC and plays a critical role in the QTP carbon cycle^47^. Permafrost degradation is predicted to generate a shift in the dominant soil weathering process, which may then facilitate greater formation of secondary carbonates in soils that are capable of sequestering atmospheric CO_2_ through a series of chemical reactions. A previous study reported that alkaline soils are capable of sequestering CO_2_ through inorganic, abiotic processes and that the amount of CO_2_ absorbed through this mechanism is determined by the salinity, alkalinity, temperature, and water content of saline or alkaline soils^48^. Soil salinization of grasslands was widespread in the Shule River Basin. Determining the CO_2_ absorption capacity of grasslands’ alkaline soils in the study area is a target for future studies.

Site variations in SIC density along environmental gradients differ from those of SOC. SIC density at depths of 0–50 cm were positively correlated with the soil clay fraction (Table 1) because low soil clay fractions may enhance carbonate leaching^47^. Generally, lithogenic and pedogenic carbonates are the main forms of SIC present in soils^49^. Lithogenic carbonate is inherited from parent material and pedogenic carbonate results from the precipitation of carbonate ions derived from root and microbial respiration that combines with calcium and magnesium ions produced in weathering reactions^50^. Accumulation of pedogenic carbonates also tends to occur in semiarid or arid areas of grassland and shrub vegetation because water deficits significantly constrain leaching^51^. Thus, the high soil clay fractions (or low soil sand and silt fractions) in our study area may decrease leaching and increase retention of pedogenic carbonates to produce the positive relationship observed between soil clay fractions and SIC. Generally, terrain topographic factors could drive the change of soil microclimate environments (water and heat), and affect the distribution of SIC. In addition, we did not consider the effects of terrain or other factors on spatial variations in SIC.

The upper reaches of the Shule River Basin belong to the permafrost transition zone in the entire QTP and have many different types of permafrost. A spatial-temporal shifts method (the spatial pattern that is represented by different types of permafrost shifting to the temporal series that is stood for different stages of permafrost degradation) can be used to discuss response characteristics of ecosystem C in the entire stages of permafrost degradation. This study region can be regarded as the latter stage of the permafrost degradation in the hinterland of QTP, because it has a relatively deep active layer thickness. In this stage, the plant species change and soil salinization was existed in the alpine steppes as permafrost degradation, which should be considered in the related simulation study. The results could also provide a basic data for the ecological environment protection of the Qilian Mountain National Nature Reserve and the entire QTP.

### Soil respiration characteristics and controls with declines in permafrost stability

Extensive and rapid retreat and thinning of permafrost could accelerate biogeochemical processes; this could result in net sources of atmospheric carbon, creation of a positive feedback, and an increase in warming^5^. SR is a key ecosystem process that releases carbon from the soil in the form of CO_2_. The resulting daily mean SR value (2.82 μmol CO_2_ m^−2^ s^−1^) was lower than that found in a large-scale investigation of the QTP (3.92 μmol CO_2_ m^−2^ s^−1^) during the summer^30^. The SR in alpine meadows first increased and then decreased from the substable permafrost zone to the unstable permafrost zone (Fig. 4), while the SR in alpine steppes first increased and then decreased from the transitional permafrost zone to the extremely unstable permafrost zone (Fig. S5). Significant correlations were found between BPC, SM, MAAT, and MAP with SR (Table 1), thus indicating that the amount of SR was controlled by BPC, SM, MAAT, and MAP in the study area. If we do not consider MAP, SM accounted for most of the spatial variation in SR according to the stepwise linear regression analysis. Permafrost degradation patterns have direct and indirect impacts on SM conditions. These patterns consequently affect SR in the Shule River Basin.

## Conclusions

Our findings suggest that permafrost degradation can lead to ecosystem carbon reduction in alpine meadows in the upper reaches of the Shule River Basin, but it was uncertain in alpine swamp meadows and alpine steppes. Owing to the influence of vegetation succession and soil salinization, trends in the amounts of SIC, SOC, APC, and BPC exhibited complex behaviors with decreasing permafrost stability in each soil or grassland type. Our results suggest that SM and soil clay fractions play a critical role in determining the spatial distribution of different carbon densities in permafrost regions of the QTP, and they should be considered as basic and effective variables for modeling or predicting carbon density changes as permafrost degradation in the future. The SR decreases were complex for the given soil or grassland type with decreasing permafrost stability, and the SR was influenced by both factors of MAP and SM. Those results could provide basic data for studying the impact of permafrost degradation on the ecosystem carbon in the entire QTP and other regions.

## Materials and Methods

### Study area description

**Figure 5:**
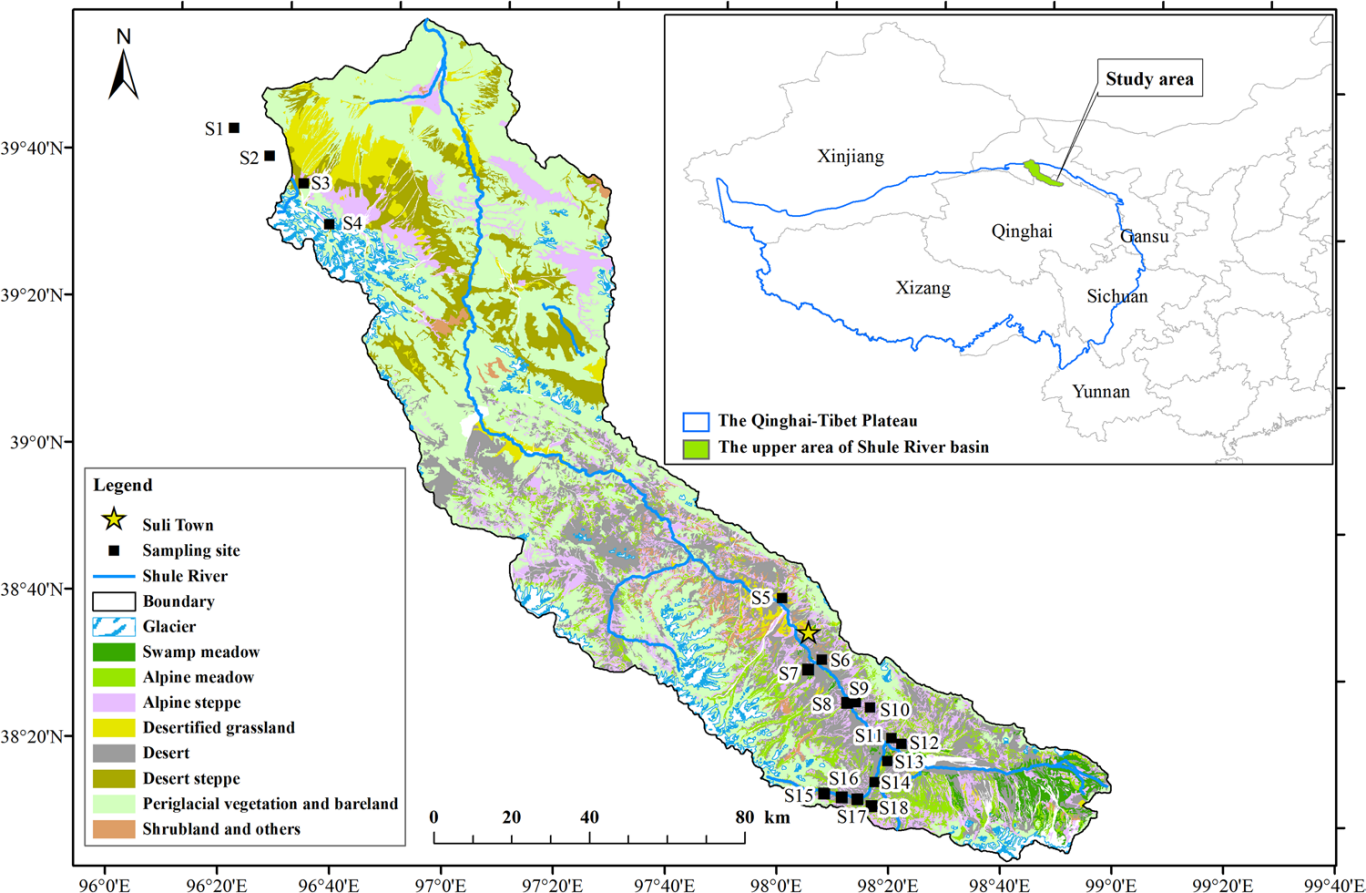
Locations of the 18 sampling sites in the upper reaches of the Shule River Basin on the northeastern margin of the QTP. The maps were laid in different data layers and were edited and generated with ESRI ArcGIS ver. 10.2.2, http://www.esri.com/.

The study was conducted in the upper reaches of the Shule River Basin (96.2°E −99.0°E, 38.2°N –40.0°N, 2,100–5,750 m a.s.l.) in the northeastern margin of the QTP (Fig. 5). The climate in this region is characterized as continental arid desert and is strongly influenced by the Asian monsoon system, whose effects decrease westward along the plateau^38^. The area has low average annual temperatures (from −4.0 °C to −19.4 °C) and precipitation (from 200 to 400 mm)^13^. Precipitation is concentrated between May and September^52^. The major grassland types include alpine swamp meadows, alpine meadows, and alpine steppes^13^. Alpine swamp meadows, alpine meadows, and alpine steppes cover areas of 227, 803, and 1,794 km^2^, respectively^38^. There were total seven main soil types in this area^38^.

Permafrost covers ~73% of the Shule River Basin^33^. The average active layer thickness of highly stable, substable, transitional, and unstable permafrost ranges from 1.5 to 3.9 m. To understand permafrost development in typical topographic locations, 20 boreholes were drilled during the summers of 2008 and 2009, which included 19 shallow boreholes (8–15 m) and one deep borehole (up to 39 m). A string of thermistors with a resolution of ±0.05 °C was used to manually measure ground temperatures at different depths of the boreholes. The distribution of high-altitude permafrost is distinct from that of high-latitude permafrost, and ground temperature has been found to be a suitable indicator of high-altitude permafrost boundaries^53^. Permafrost stability was obtained from a permafrost distribution model that used an equivalent elevation method based on 90 m grid-DEM data and 15 m depth ground temperature of boreholes^32^. The equivalent elevation method procedures were described by Li *et al*.^32^, and the modeling results were expanded to the upper reaches of the Shule River Basin. We identified six types of permafrost with various degrees of stability based on the mean annual ground temperature (MAGT) values at a depth of 15 m. These types include highly stable permafrost (MAGT < −5.0 °C), stable permafrost (−5.0 °C < MAGT < −3.0 °C), substable permafrost (−3.0 °C < MAGT < −1.5 °C), transitional permafrost (−1.5 °C < MAGT < −0.5 °C), unstable permafrost (−0.5 °C < MAGT < 0.5 °C), and extremely unstable permafrost (MAGT > 0.5 °C)^13,54^. The distribution of permafrost is shown in Fig. S7.

### Field surveys, sample collection, and analysis

Based on previous field investigations of three grassland areas^13^, we selected 18 sites (Table S1) using a quadrat survey and samples collected in July and August 2013. We inserted a polyvinyl chloride (PVC) collar (13 cm internal diameter, 10 cm height) 3–5 cm into the soil for SR measurements in 12 of the selected sites. Live plants inside the soil collar were removed before measurement to eliminate the effects of aboveground plant respiration. We used a LI-8100 portable soil CO_2_ flux system (Li-Cor Inc., Lincoln, NE, USA) for SR measurements^55^. Measurements were taken by placing the LI-8100 chamber on the PVC collars for 1–2 min at 3-min intervals for 2 h at each site. To obtain the diurnal pattern, we also measured the complete diurnal variation of SR at two sites (Fig. S2). We then calculated the ratios of instant SR at sampling time to the daily mean SR for the two sites. Using these ratios, we calculated the daily mean SR of non-diurnal sites. We measured soil temperatures (°C) at a depth of 5 cm adjacent to each PVC collar using a thermocouple probe (Li-8100-201) connected to the LI-8100 during each SR measurement.

Three quadrats (50 cm × 50 cm) at each sampling site were randomly chosen, from which we harvested living aboveground phytomass and litter. The aboveground phytomass included living aboveground phytomass and litter. Subsequently, we collected belowground phytomass and soil samples from each quadrat by combining five 4.8 cm diameter soil cores obtained in an X-shaped pattern, which were divided into sections of 0–10 cm, 10–20 cm, 20–30 cm, 30–40 cm, and 40–50 cm. At each site, three additional soil sample replicates of equal volume were collected from each soil depth using a cutting ring (100 cm^3^ volume) to determine soil bulk density. In addition, soil temperature and bulk electrical conductivity (Ecb) were measured using a WET-2 sensor.

Belowground phytomass samples were crushed manually, passed through a 0.2 cm sieve after removing impurities, and then repeatedly cleaned in water. The aboveground phytomass (including live aboveground phytomass and litter) and all belowground phytomass material were dried at 80 °C and weighed to obtain phytomass quantities. We gravimetrically measured SM on fresh soil samples after drying them at 105 °C for 24 h. Soil total carbon (TC, %) and TN (%) were measured using a vario MACRO cube elemental analyzer (Elementar, Germany). We analyzed SOC using the modified Walkley–Black method, which comprised boiling a soil-dichromate-sulfuric acid mixture for 5 min at 175 °C^56^. Soil particle size distributions were determined by wet sieving^57^.

### Data collection and analysis

The vegetation type data set (Fig. 5) was provided by the Environmental and Ecological Science Data Center for West China, National Natural Science Foundation of China (http://westdc.westgis.ac.cn). We obtained MAAT and MAP datasets from climate data in the study area^38^. The active layer thickness data were derived using Pulse EKKO PRO ground-penetrating radar measurement data from 2009 and borehole drilling data from 2010^13^. We calculated SIC based on the difference between TC (%) and SOC (%) using the equation [SIC_i_ = 0.96 × (SC_i_ − SOC_i_) + 0.69], which was adopted to eliminate errors involved in SOC measurements in the grasslands of the QTP^47^. We calculated APC and BPC densities from the aboveground and belowground phytomass data using a *K*_*A*_ factor of 0.45 and a *K*_*B*_ factor of 0.40, respectively^58^. Total PC is the sum of APC and BPC in the top 50 cm. To determine the vertical distributions of SIC and SOC densities, we quantified these values in five, 10 cm intervals in the upper 50 cm in each profile. The procedures for carbon density calculations are described in a previous study^38^. SC density was determined as the sum of SIC and SOC densities in the top 50 cm, while the EC density is the sum of SIC, SOC, APC, and BPC densities. The values for additional soil properties (SM, ST, Ecb, and soil clay fraction) were taken as the average across depths of 0–50 cm, which were then used in statistical analyses. The different forms of carbon in cold calcic soils (Kastanozems), alpine swamp meadows, alpine meadows, and alpine steppes were selected to study the variation among the different permafrost stability zones. We conducted a one-way analysis of variance (Duncan’s multiple range test) to evaluate differences in the carbon densities in different permafrost types. We conducted linear regression and correlation analyses using SPSS software (version 16.0) to characterize the relationship between abundances of different forms of carbon and MAAT, MAP, and other soil parameters. We considered a P-value of *P* < 0.05 to be significant.

## Electronic supplementary material

Supporting information
