# Supplementary material for: The effect of decreasing permafrost stability on ecosystem carbon in the northeastern margin of the Qinghai–Tibet Plateau
Source: Sci Rep. 2018 Mar 8;8:4172. doi: 10.1038/s41598-018-22468-6 (PMC5843650; doi:10.1038/s41598-018-22468-6)
Supplement: Supplementary file 1 — Supporting information [file 41598_2018_22468_MOESM1_ESM.pdf]

Scientific Reports

Supporting Information for

The effect of decreasing permafrost stability on ecosystem carbon in the northeastern margin of the Qinghai–Tibet Plateau

Wenjie Liu [1, 2], Shengyun Chen [2], Junyi Liang [3], Xiang Qin [2], Shichang Kang [2], Jiawen Ren [2], and Dahe Qin [2]

[1] Institute of Tropical Agriculture and Forestry, Hainan University, Haikou, 570228, China

[2] Qilian Shan Station of Glaciology and Ecologic Environment, State Key Laboratory of Cryospheric Science, Northwest Institute of Eco-Environment and Resources, Chinese Academy of Sciences, Lanzhou, 730000, China

[3] Environmental Science Division and Climate Change Science Institute, Oak Ridge National Laboratory, Oak Ridge, Tennessee 37831, USA

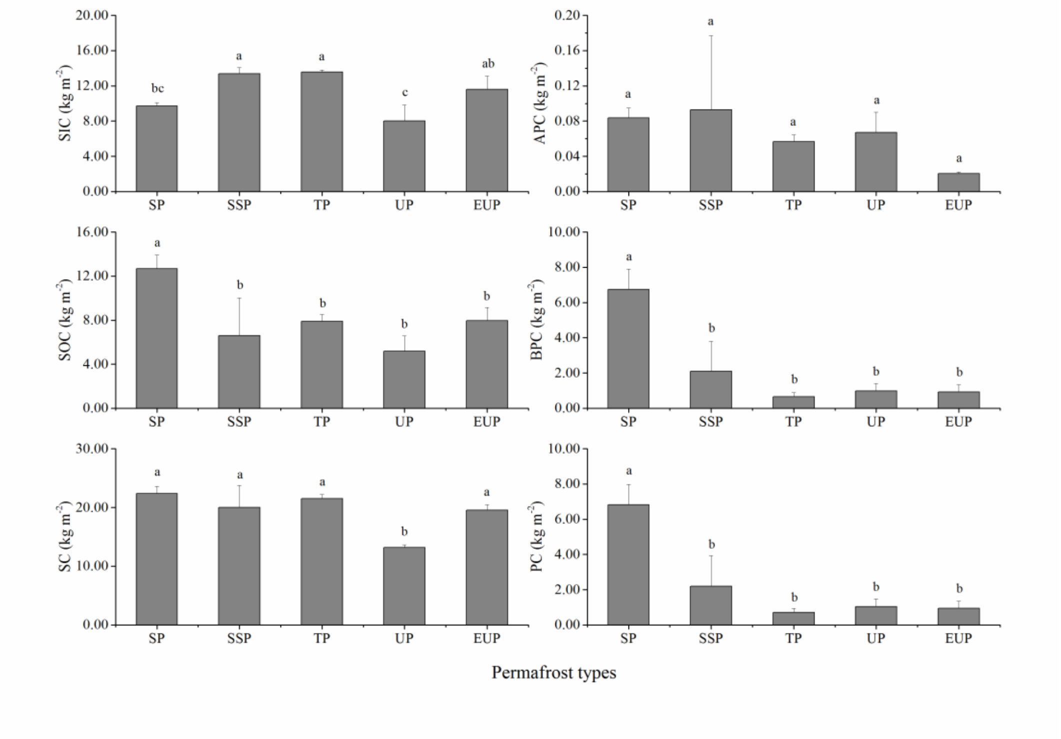

Figure S1 Variations in soil organic carbon (SOC), soil inorganic carbon (SIC), soil carbon (SC, the sum of SIC and SOC), and belowground phytomass carbon (BPC) at depths of 0–50 cm, along with above ground phytomass carbon (APC) and total phytomass carbon (PC, the sum of BPC and APC) among different permafrost zones in cold calcic soils (SP: stable permafrost; SSP: sub-stable permafrost; TP: transitional permafrost; UP: unstable permafrost; EUP: extremely unstable permafrost). Error bars express the standard deviation from the mean. Within one form carbon, different permafrost zones show significant differences when columns are marked with different letters and non-significant differences when columns are marked with the same letters. The figures were generated using OriginPro 2016 (64-bit) b9.3.226 (http://www.originlab.com/).

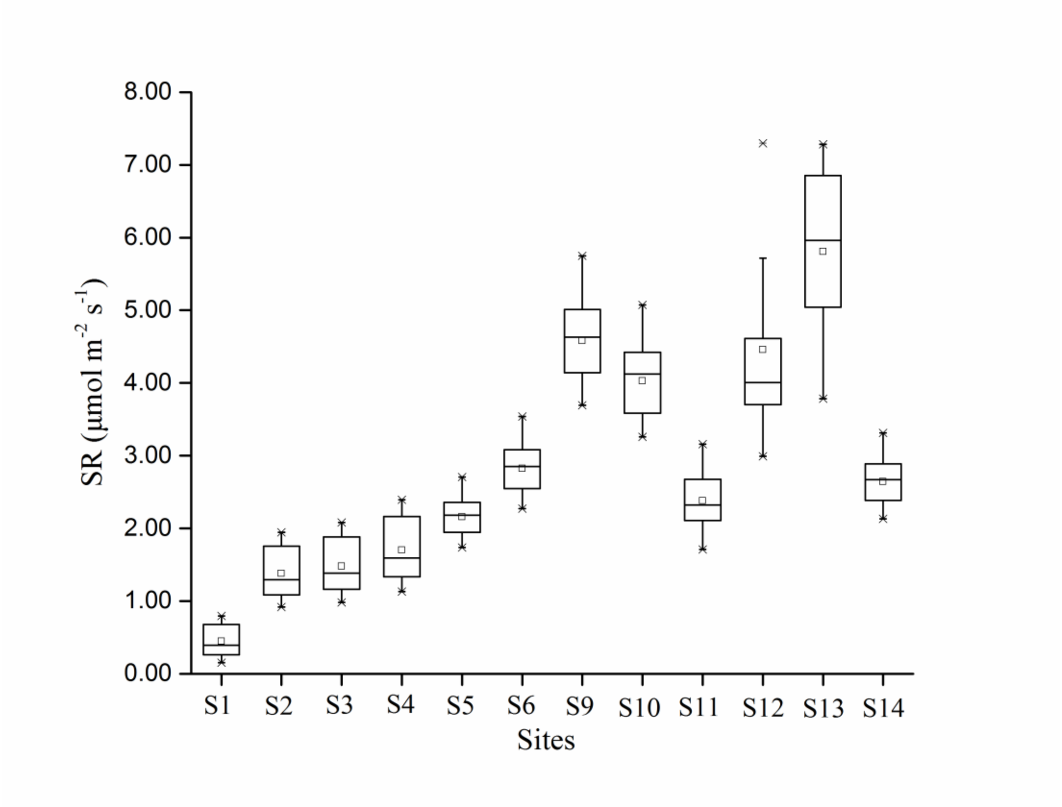

Figure S2 Box and whisker plots of mean daily soil respiration (SR) at 12 sampling sites. The figure was generated using OriginPro 2016 (64-bit) b9.3.226 (http://www.originlab.com/).

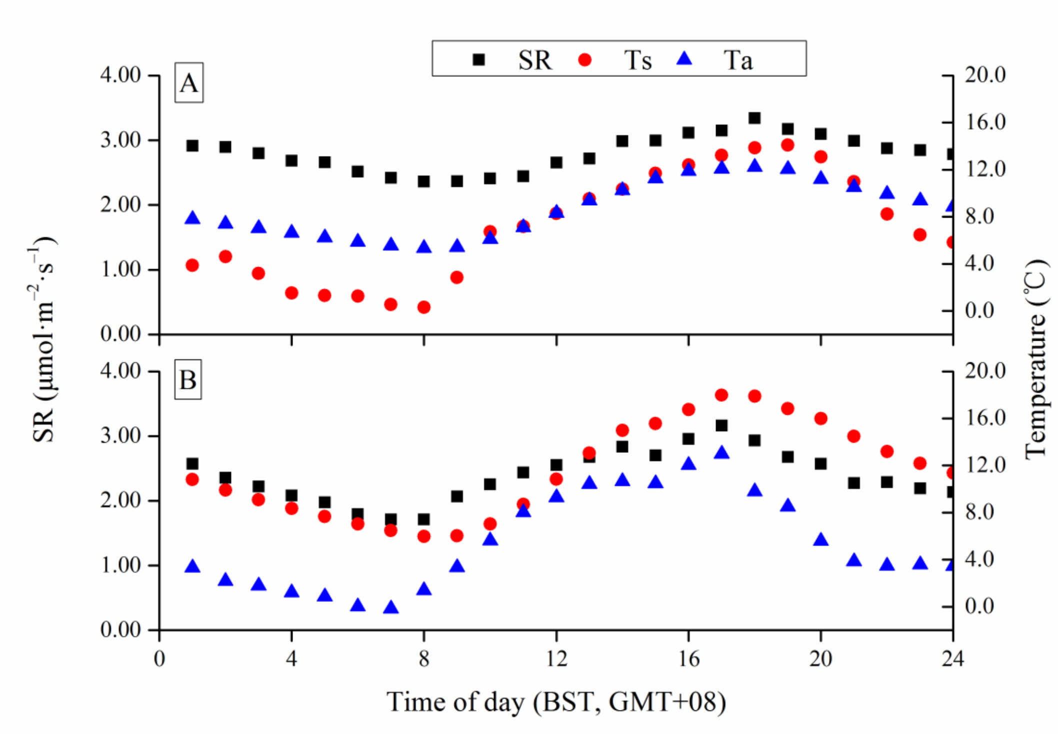

Figure S3 Diurnal variations in soil respiration (SR), soil temperature (Ts), and air temperature (Ta) at sites SLP2 (A) and SLP4 (B). The figure was generated using OriginPro 2016 (64-bit) b9.3.226 (http://www.originlab.com/).

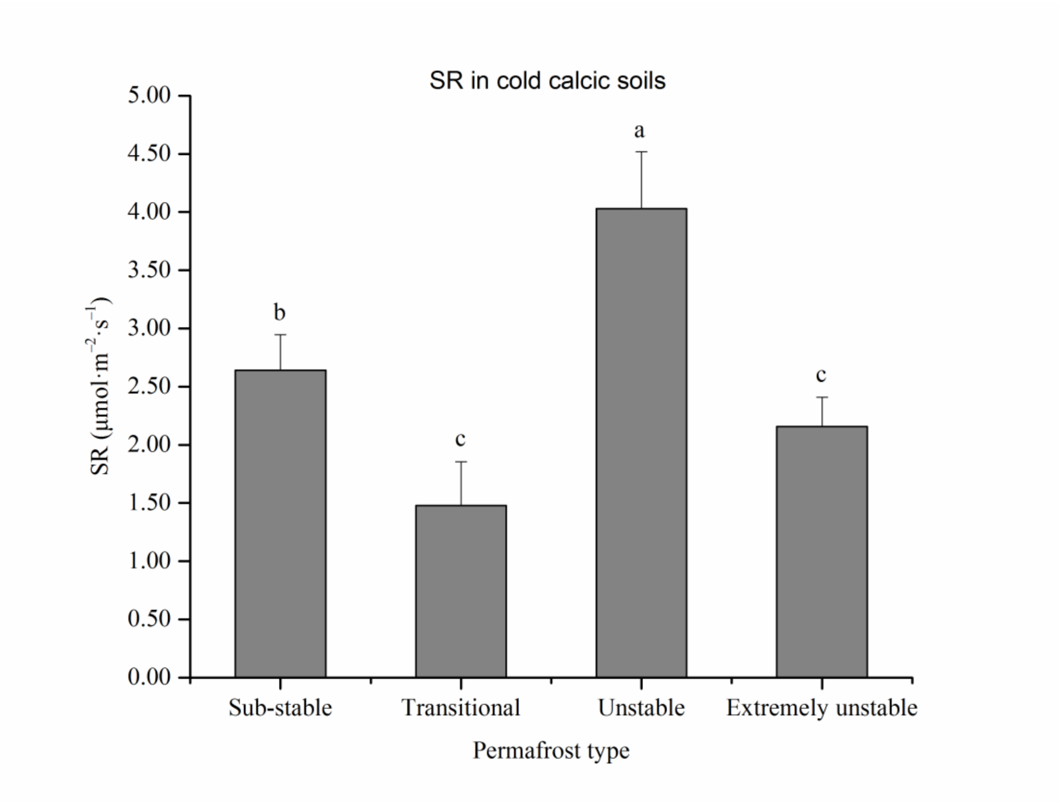

Figure S4 Variations in soil respiration (SR) among different permafrost zones in cold calcic soils. Error bars express the standard deviation from the mean. Different permafrost zones show significant differences of SR when columns are marked with different letters and non-significant differences of SR when columns are marked with the same letters. The figure was generated uing OriginPro 2016 (64-bit) b9.3.226 (http://www.originlab.com/).

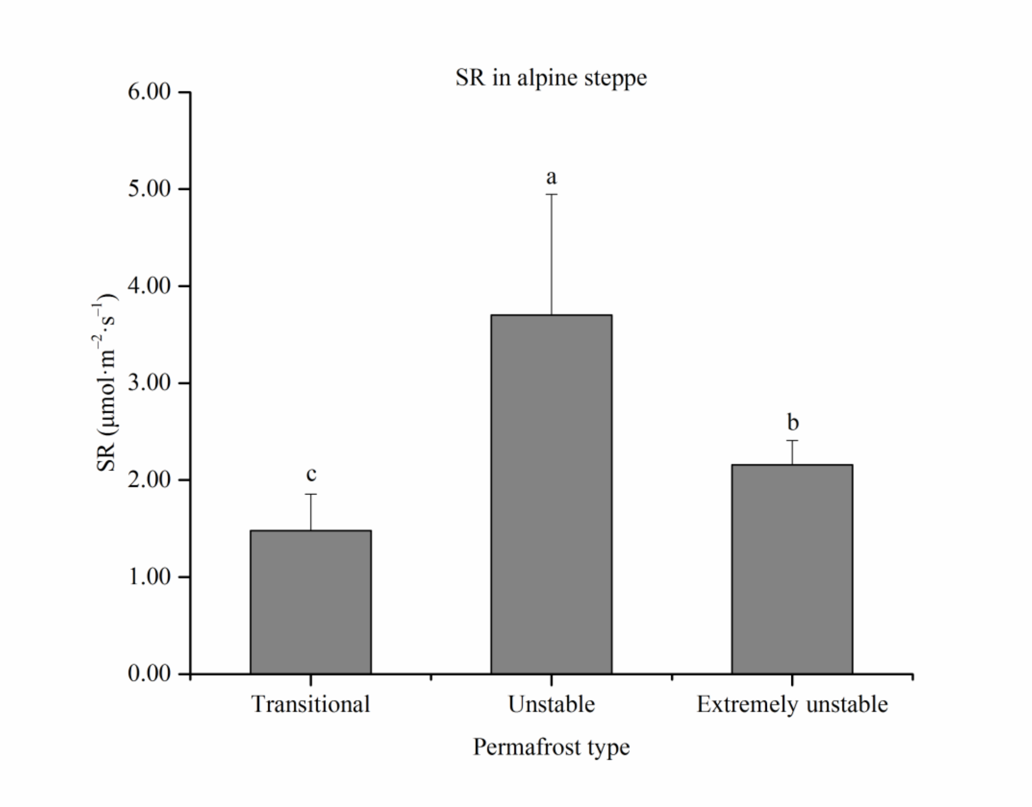

Figure S5 Variations in soil respiration (SR) among different permafrost zones in alpine steppe. Error bars express the standard deviation from the mean. Different permafrost zones show significant differences of SR when columns are marked with different letters and non-significant differences of SR when columns are marked with the same letters. The figure was generated uing OriginPro 2016 (64-bit) b9.3.226 (http://www.originlab.com/).

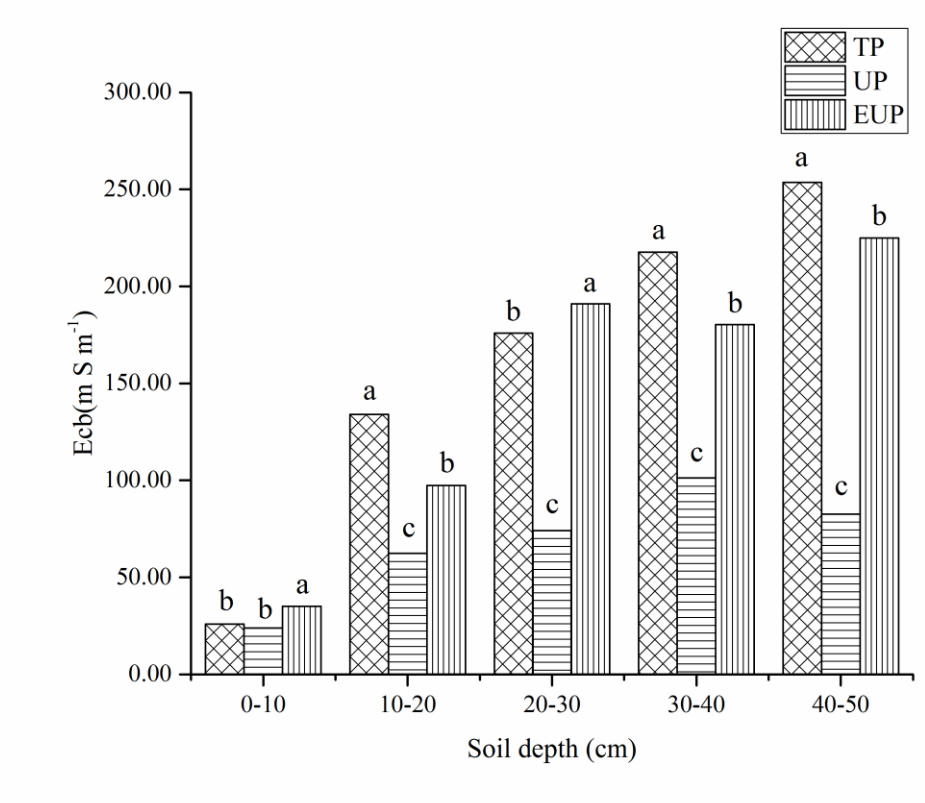

Figure S6 Variations in bulk electrical conductivity (Ecb) among different permafrost zones in alpine steppe. Different permafrost zones show significant differences of Ecb when columns are marked with different letters and non-significant differences of Ecb when columns are marked with the same letters. The figure was generated using OriginPro 2016 (64-bit) b9.3.226 (http://www.originlab.com/).

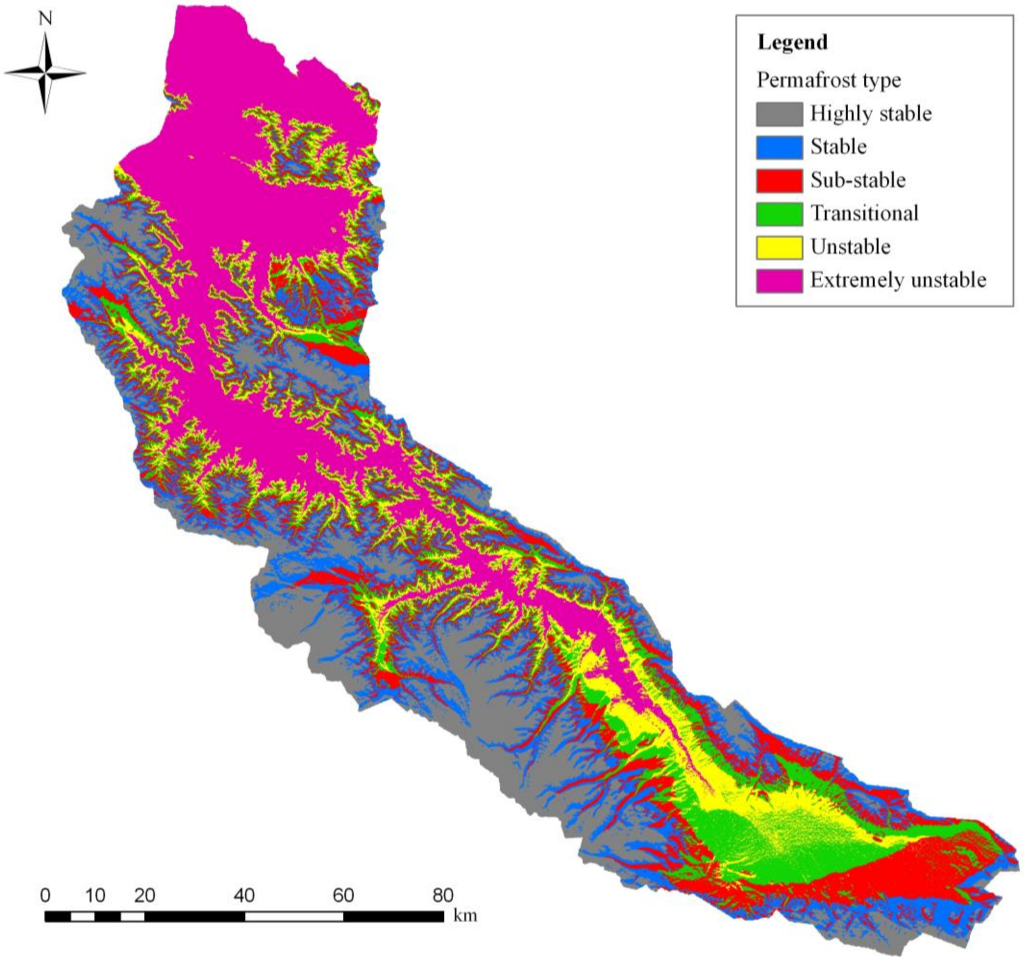

Figure S7 Distribution of the permafrost in the upper reaches of Shule River Basin. The maps were generated with ESRI ArcGIS ver. 10.2.2, http://www.esri.com/

Table S1. Number, location, dominant plants and types of vegetation sites in the upper reaches of Shule River Basin (After Chen et al. [13] and Liu et al. [38])

Site Number	Longitude/Latitude (°E / °N)	Elevation (m)	Dominant plants	Permafrost type	ALT (m)	Grassland type	Soil type
S1	96°11′24″/ 39°44′43″	2519	*Kalidium foliatum*, *Allium polyrhizum*	EUP	–[a]	D	Gray-brown desert soils
S2	96°18′26″/ 39°41′09″	3016	*Stipa sareptana var. krylovii*, *Artemisia minor*	EUP	–	DG	Brown pedocals
S3	96°24′59″/ 39°37′57″	3438	*Stipa purpurea*, *Leontopodium leontopodioides*	TP	3.5	AS	Cold caclic soils
S4	96°30′09″/ 39°32′13″	4105	*Saussurea nigrescens*, *Potentilla potaninii*	H-SP	1.6	PV	Frigid caclic soils
S5	97°57′32″/ 38°46′32″	3636	*Stipa basiplumosa*, *Limonium aureum var. dielsianum*	EUP	–	AS	Cold caclic soils
S6	98°05′37″/ 38°38′11″	3750	*Poa pratensis*, *Stipa basiplumosa*	UP	2.4	AS	Frigid caclic soils
S7	98°3′15″/ 38°37′10″	3738	*Stipa*, *Potentilla potaninii*	UP	2.3	AS	Frigid caclic soils
S8	98°10′41″/ 38°33′0″	3806	*Stipa basiplumosa*, *Leontopodium alpinum*	UP	2.0	AS	Frigid caclic soils
S9	98°12′20″/ 38°33′02″	3832	*Stipa purpurea*, *Artemisia minor*	UP	2.9	AS	Frigid caclic soils
S10	98°14′49″/ 38°32′29″	3936	*Stipa purpurea*, *Artemisia nanschanica*	UP	2.7	AM	Cold caclic soils
S11	98°19′25″/ 38°28′33″	3890	*Carex moorcroftii*, *Stipa purpurea*	UP	3.9	AM	Frigid caclic soils
S12	98°20′51″/ 38°27′59″	3863	*Kobresia tibetica*, *Carex parva*	TP	1.8	AMM	Bog soils
S13	98°18′31″/ 38°25′16″	3882	*Kobresia capillifolia*, *Carex moorcroftii*	TP	2.1	AM	Felty soils
S14	98°16′14″/ 38°21′17″	4014	*Kobresia pygmaea*, *Kobresia humilis*	SSP	2.3	AM	Cold caclic soils
S15	98°7′42″/ 38°20′26″	4191	*Carex moorcroftii*, *Kobresia humilis*	SP	1.7	AM	Felty soils
S16	98°10′51″/ 38°20′2″	4099	*Potentilla potaninii*, *Corydalis dasyptera*	SSP	2.0	BSBG	Cold caclic soils
S17	95°13′44″/ 38°19′58″	4036	*Kobresia pygmaea*, *Kobresia tibetica*	SP	1.5	AM	Cold caclic soils
S18	98°16′19″/ 38°19′7″	4067	*Kobresia tibetica*, *Carex moorcroftii*	SSP	1.7	AMM	Bog soils

D: desert, DG: desertified grassland, AS: alpine steppe, PV: periglacial vegetation, AM: alpine meadow, AMM: alpine marsh meadow, BSBG: "black-soil-beach" grassland; H-SP: highly stable and stable permafrost, SSP: sub-stable permafrost, TP: transitional permafrost, UP: unstable permafrost, EUP: extremely unstable permafrost, ALT: active layer thickness. a: no data for EUP, because EUP is equal to seasonal frozen soil.

Table S2 One-sample Kolmogorov-Smirnov test for variables

	SIC (kg m^{-2})	SOC (kg m^{-2})	APC (kg m^{-2})	BPC (kg m^{-2})	EC (kg m^{-2})	TN (kg m^{-2})	SM (%)	Ecb (mS m^{-1})	ST (℃)	Clay (%)	MAAT (℃)	MAP (mm)	SR (n = 12) (μmol m^{-2} s^{-1})
Kolmogorov-Smirnov Z	0.55	1.16	0.72	1.33	0.87	1.07	0.88	1.29	0.49	0.63	1.06	1.00	0.54
Asymp. Sig.(2-tailed)[a]	0.93	0.13	0.68	0.06	0.43	0.20	0.42	0.07	0.97	0.83	0.21	0.27	0.93

[a]: Asymp. Sig. (2-tailed)>0.05, means the data of variable is normal distribution.

References

13. Chen, S. et al. Response characteristics of vegetation and soil environment to permafrost degradation in the upstream regions of the Shule River Basin. *Environmental Research Letters* 7, 045406 (2012).

38. Liu, W. et al. Storage, patterns, and control of soil organic carbon and nitrogen in the northeastern margin of the Qinghai–Tibetan Plateau. *Environmental Research Letters* **7**, 035401 (2012).
